# From Pseudocyst to Pseudoaneurysm: A Complex Pancreatic Vascular Emergency Managed With a Multi-stage Interventional Radiology Approach

**DOI:** 10.7759/cureus.88448

**Published:** 2025-07-21

**Authors:** Upali Anand, Evangelia Florou, Andrea Fernandez-Pujol, Nabil Kibriya, Matthew J Seager, Evangelos Prassas

**Affiliations:** 1 Hepato-Pancreato-Biliary Surgery, King's College Hospital, London, GBR; 2 Hepato-Pancreato-Biliary Interventional Radiology, King's College Hospital, London, GBR; 3 General Surgery, King's College Hospital, London, GBR

**Keywords:** chronic pancreatitis, embolization, gastroduodenal artery, glubran ii, interventional radiology, pancreaticoduodenal arcade, pancreaticoduodenal artery, pancreatic pseudocyst, pseudoaneurysm, superior mesenteric artery

## Abstract

Acute-on-chronic pancreatitis is frequently complicated by the formation of pancreatic pseudocysts (PPs). Haemorrhage within a pseudocyst is a life-threatening event, typically resulting from the development of a pseudoaneurysm (PA) involving adjacent arterial structures. First-line treatment is endovascular embolization, an interventional radiology (IR) approach. However, if embolization fails, remaining treatment options are severely limited, as surgery is often considered high-risk or unfeasible. We present a case of chronic pancreatitis complicated by a haemorrhagic PP complicated by PA formation of several adjacent vessels within the pancreatic head. The patient was successfully managed with multiple embolization sessions, performed by IR, the only viable treatment option in this scenario.

A 43-year-old man with a history of alcohol-related liver cirrhosis (Child-Pugh B), alcoholic hepatitis, and recurrent chronic pancreatitis presented with melaena. Computed tomography (CT) imaging revealed a pseudocyst in the pancreatic head containing internal haemorrhage, with active bleeding from multiple arterial feeders within the pancreaticoduodenal arcade (PDAA). Five direct angiography sessions were required. A large amount and combination of coils as well as large volumes of glue (Glubran II; GEM S.r.l., Lucca, Italy), and stent placement were all necessary to achieve complete occlusion of all feeding vessels causing bleeding within the PP. Surgical intervention would have necessitated a pancreaticoduodenectomy in a hostile operative field, with prohibitively high morbidity and mortality.

Endovascular embolization remains the treatment of choice for PA in the setting of haemorrhagic pancreatic pseudocysts. This case highlights the life-saving role of IR, particularly when surgical options are unfeasible. It also emphasizes the importance of timely escalation, multidisciplinary decision-making, and advanced embolization techniques.

Notably, this is the first reported case of a persistently haemorrhagic PP successfully managed with a multi-stage interventional radiology approach incorporating Glubran II, coils, thrombin, and covered stent deployment. Given the rarity of the condition and the effectiveness of the treatment, this case describes an evolving endovascular strategy finding application in a highly complex clinical scenario.

## Introduction

Chronic pancreatitis results from recurrent inflammation of the pancreas and may lead to complications such as pancreatic pseudocyst (PP) formation, thrombosis of peripancreatic venous structures, and pseudoaneurysm (PA) development [1-3]. PA formation ranges from 4-10% of chronic pancreatitis cases and can be fatal, with rupture-associated mortality rates reaching 15-40% [2,4].

Pseudocyst formation is common in both acute and acute-on-chronic pancreatitis, with reported bleeding incidence ranging from 3.2% to 17% [1,2,4]. Treatment options for haemorrhagic PP include primarily embolization of the affected vessel via interventional radiology (IR) [2,4]. Untreated haemorrhagic PP has a mortality reaching 90% [1], however, even after treatment the mortality remains in a range of 18-29% [1-3].

Completion of treatment with surgery is not considered necessary, but very seldom may be indicated [1]. On the other hand, emergency surgery for bleeding PP carries high mortality rates reaching up to 45% in the literature [1,4,5].

We describe a rare and technically demanding case that underlines both the life-threatening potential of haemorrhagic PP and the pivotal role of advanced IR in achieving definitive management.

## Case presentation

**Figure 1:**
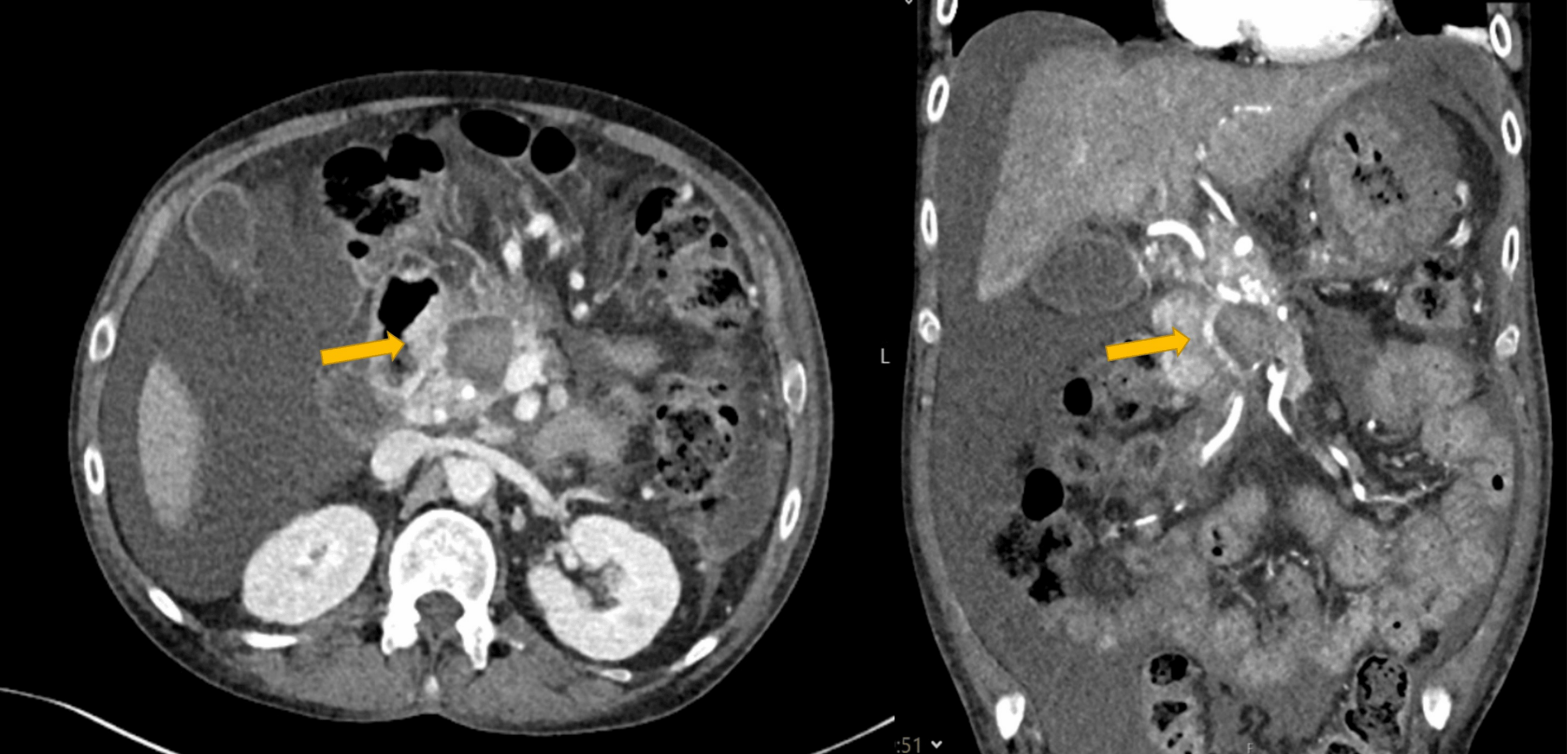
Haemorrhagic PP managed with multi-session angiographic embolization to achieve haemostasis. Axial and coronal view of contrast enhanced CT scan demonstrating the initial appearance of PP (yellow arrows) in this case which is not filling with contrast at this early stage. PP: Pancreatic pseudocyst; CT: computed tomography

A 43-year-old male presented with melena, associated with jaundice and abdominal pain. He had a background of alcohol-related liver cirrhosis with portal hypertension, alcohol-induced chronic pancreatitis with multiple episodes in the past causing insulin-dependent diabetes mellitus. At the time of presentation, he reported active alcohol consumption, however, he was also engaged with the local alcohol cessation services. On this first admission, he was treated for decompensated liver cirrhosis as was clinically found encephalopathic with deranged liver biochemistry. The gastrointestinal (GI) bleeding was investigated with upper GI endoscopy, which revealed grade 1 lower oesophageal varices, portal hypertensive gastropathy, and possible ectopic varices in the first part of the duodenum, findings to which the episode of melena was attributed. However, there were no overt signs of recent bleeding on endoscopy. A computed tomography (CT) showed features of chronic pancreatitis with a small PP, measuring 34x46x26mm, lying at the pancreatic head with no signs of internal haemorrhage (Figure 1). He received a blood transfusion and a course of antibiotics and was discharged. 

Three months later, he re-presented reporting episodes of melena for the past two days and laboratory findings showed a significant haemoglobin drop to 65g/dL. He remained haemodynamically stable during this second admission, received five units of packed red blood cells in total and the upper GI endoscopy was repeated. The study once again showed oesophageal varices with no signs of recent bleeding. A CT angiogram revealed that the previously noted PP had increased in size and had developed signs of internal haemorrhage. The PP, which was now measuring 57x57x64mm, had evolved into a blood-filled cavity with a potential source of PA formed on an arterial branch within the pancreatic head, likely arising from the pancreaticoduodenal arcade (PDAA).

The local IR team proceeded to a first direct angiography. Upon catheterisation of the superior mesenteric artery (SMA), rapid filling of the haemorrhagic PP was demonstrated, with the impression that this was fed primarily by the inferior pancreaticoduodenal artery (IPDA). Multiple micro-coils were deployed to the IPDA, resulting in sealing the bleeding source. At the same sitting, and due to the proximity of the PP to the gastroduodenal artery (GDA), the latter was also embolised as a preventative measure of further bleeding and PA formation in the area.

**Figure 2:**
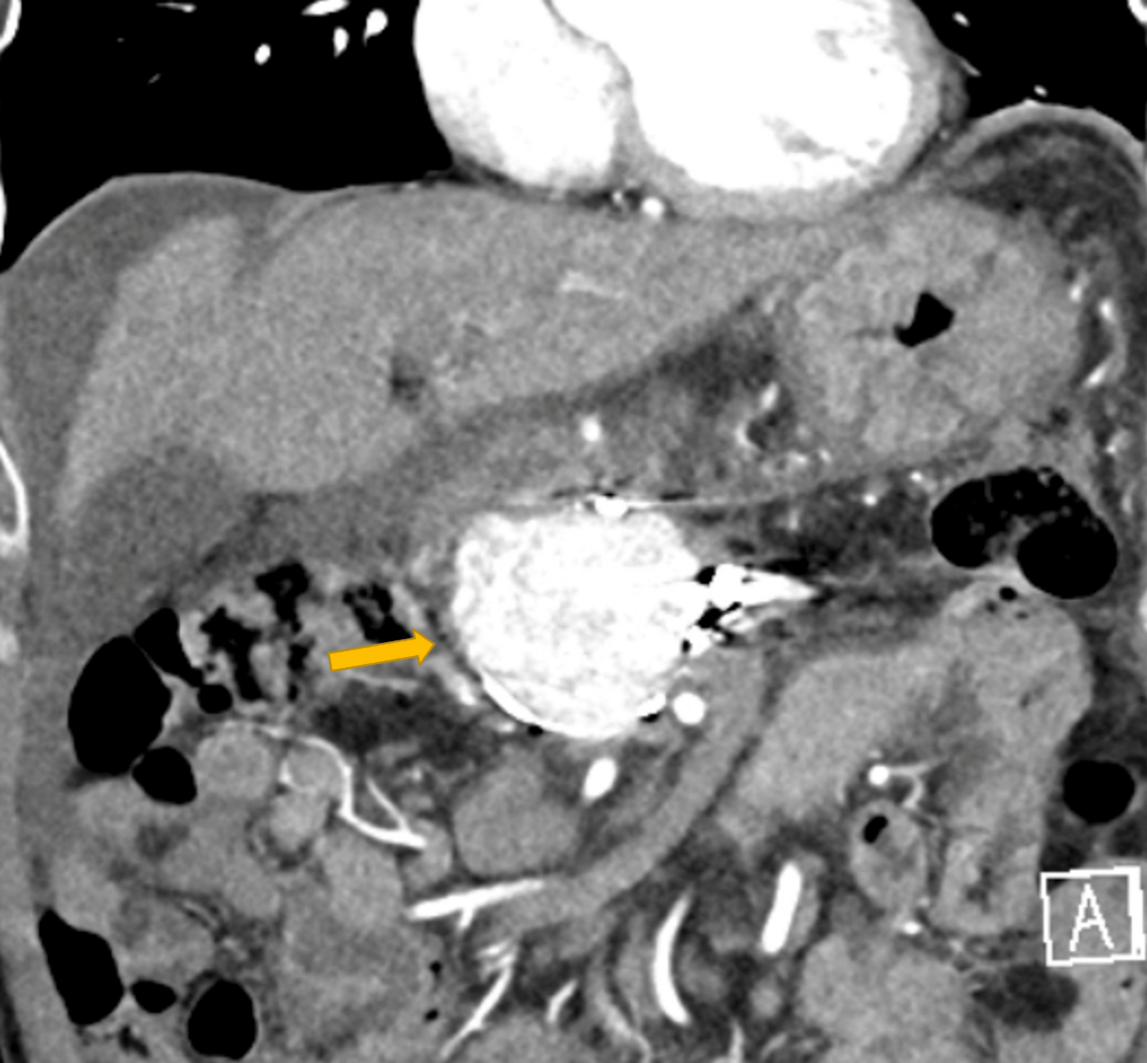
Haemorrhagic PP managed with multi-session angiographic embolization to achieve haemostasis. The PP (yellow arrow) is partially embolised but still filling up with contrast on CT. PP: Pancreatic Pseudocyst; CT: computed tomography

Interestingly enough, a repeat follow-up CT scan the following day showed ongoing filling of the PP. A second angiography followed, where repeat catheterisation of the SMA confirmed persistent contrast extravasation via a small tortuous branch arising from the PDAA, which was successfully embolised. Three days later, new onset of abdominal pain prompted a repeat follow-up CT which showed that the PP was persistently filling with contrast and as local IR options were exhausted, the case was referred to our specialised centre (Figure 2).

**Figure 3:**
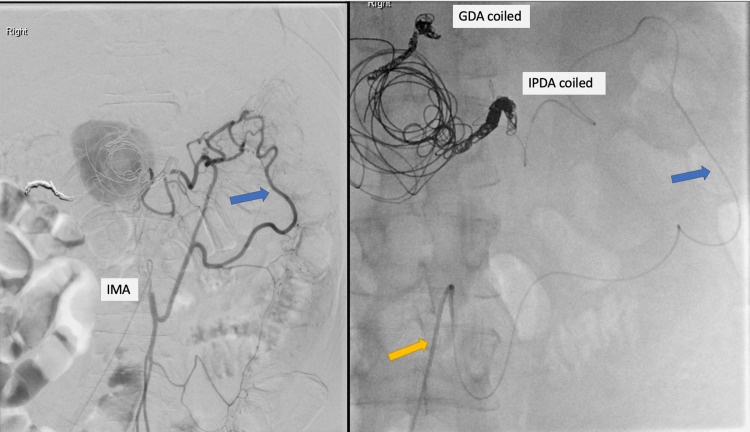
Haemorrhagic PP managed with multi-session angiographic embolization to achieve haemostasis The angiography on the left show continuation of blood filling of the PP due to communication with IMA via marginal artery (blue arrow). On the right, IMA is catheterised and guidewire (yellow arrow) accesses the marginal artery for embolization. The PP has been previously partially embolised. Previously noted route of blood filling vessel from IPDA has been coiled and GDA was pre-emptively coiled as well. PP: Pancreatic Pseudocyst; IPDA: inferior pancreatico-duodenal artery; GDA: gastro-duodenal artery; IMA: inferior mesenteric artery

The third direct angiography, performed at our specialist IR centre, revealed residual inflow to the PP from a branch communicating with the inferior mesenteric artery (IMA) (Figure 3). Embolization of the contributing vessels was performed using 2-4 mm detachable Azur CX coils (Terumo, Tustin, CA, USA) and Glubran II (Glubran II; GEM S.r.l., Lucca, Italy) mixed with Lipiodol (Guerbet, Raleigh, CA, USA) in a 1:1 ratio, resulting in cessation of filling from the IMA. The assessment was completed with ultrasonography scan (USS). The latter revealed once again persistent “yin-yang” flow within the PP, a classic radiological sign indicative of bidirectional blood flow within the area examined. Under ultrasound guidance and using a 22-gauge Chiba needle, a transabdominal direct puncture of the PP was performed. Following confirmation of relatively static flow within the cavity, 1500IU of thrombin was injected directly.

**Figure 4:**
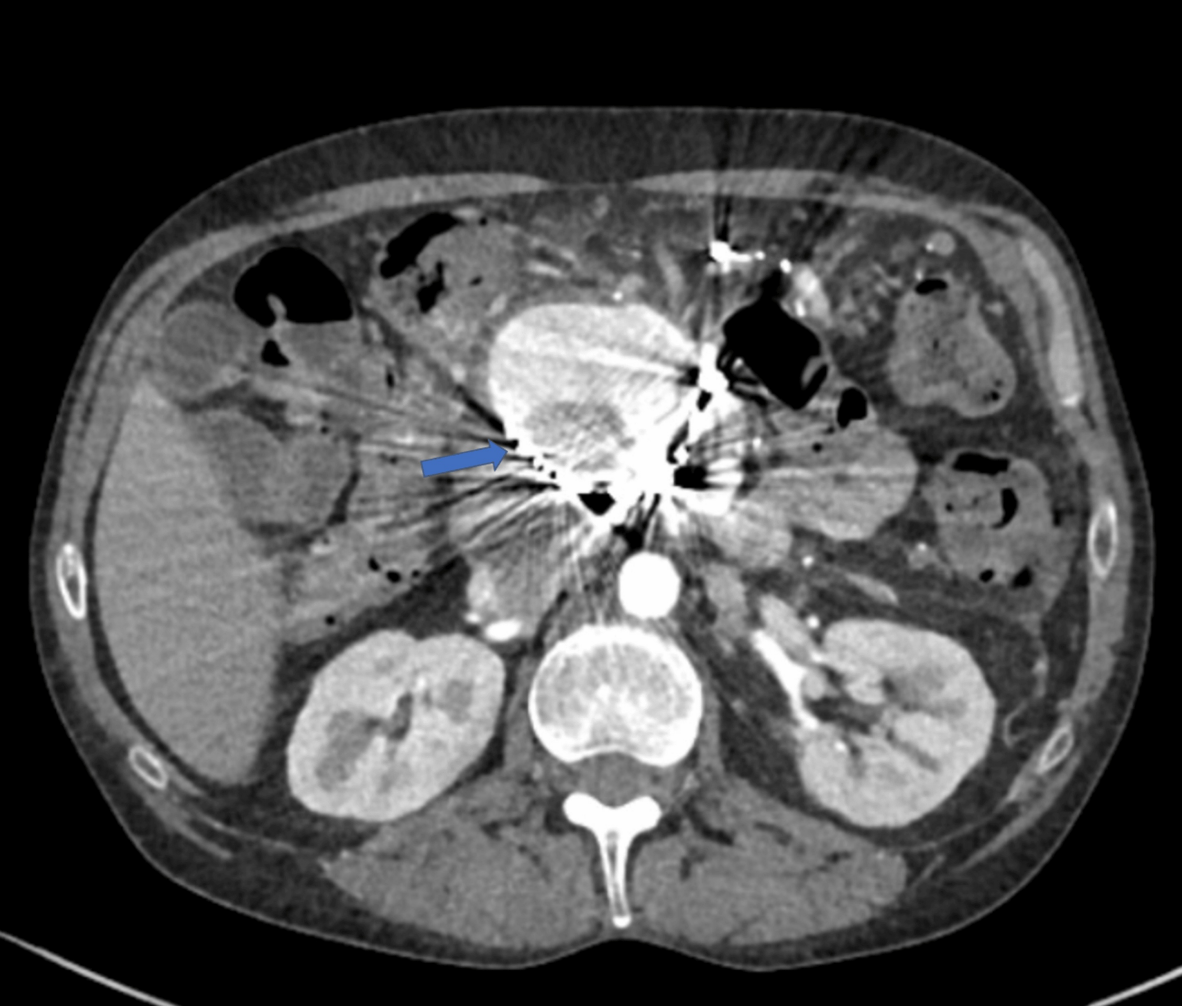
Haemorrhagic PP managed with multi-session angiographic embolization to achieve haemostasis. Ongoing filling of PP with contrast (blue arrow) evident with only partial thrombosis and ongoing communication with surrounding arterial structure. PP: Pancreatic Pseudocyst

Despite this, a follow-up CT scan performed 48 hours later demonstrated continued filling of the PP (Figure 4). A fourth angiogram again showed opacification of the PP via branches from the SMA. Super-selective catheterization of the feeding vessel could not be achieved despite multiple attempts; thus, a balloon-expandable covered stent was deployed across the origin of the SMA to occlude the feeding vessel. A completion angiogram confirmed temporary exclusion of the lesion; however, a repeat CT five days later revealed that although the PP had partially thrombosed, contrast opacification persisted.

Given the complexity of the case, it was presented at the multidisciplinary team (MDT) meeting. Surgery was deemed extremely high-risk due to the hostile surgical field, characterized by chronic inflammation, proximity to major vessels, and ongoing perfusion of the PP. Concerns about uncontrollable intraoperative bleeding and significant postoperative morbidity led the MDT to recommend further IR intervention. Before reaching a treatment dead end, another radiological intervention was suggested.

A fifth angiographic procedure revealed re-filling of the PP through the previously coiled GDA, as well as suspected contribution from the dorsal pancreatic artery (DPA), originating from the splenic artery. Both arteries were selectively catheterized and embolized using Glubran II:Lipiodol in a 1:2 ratio and three 15 mm Penumbra detachable soft packing coils.

**Figure 5:**
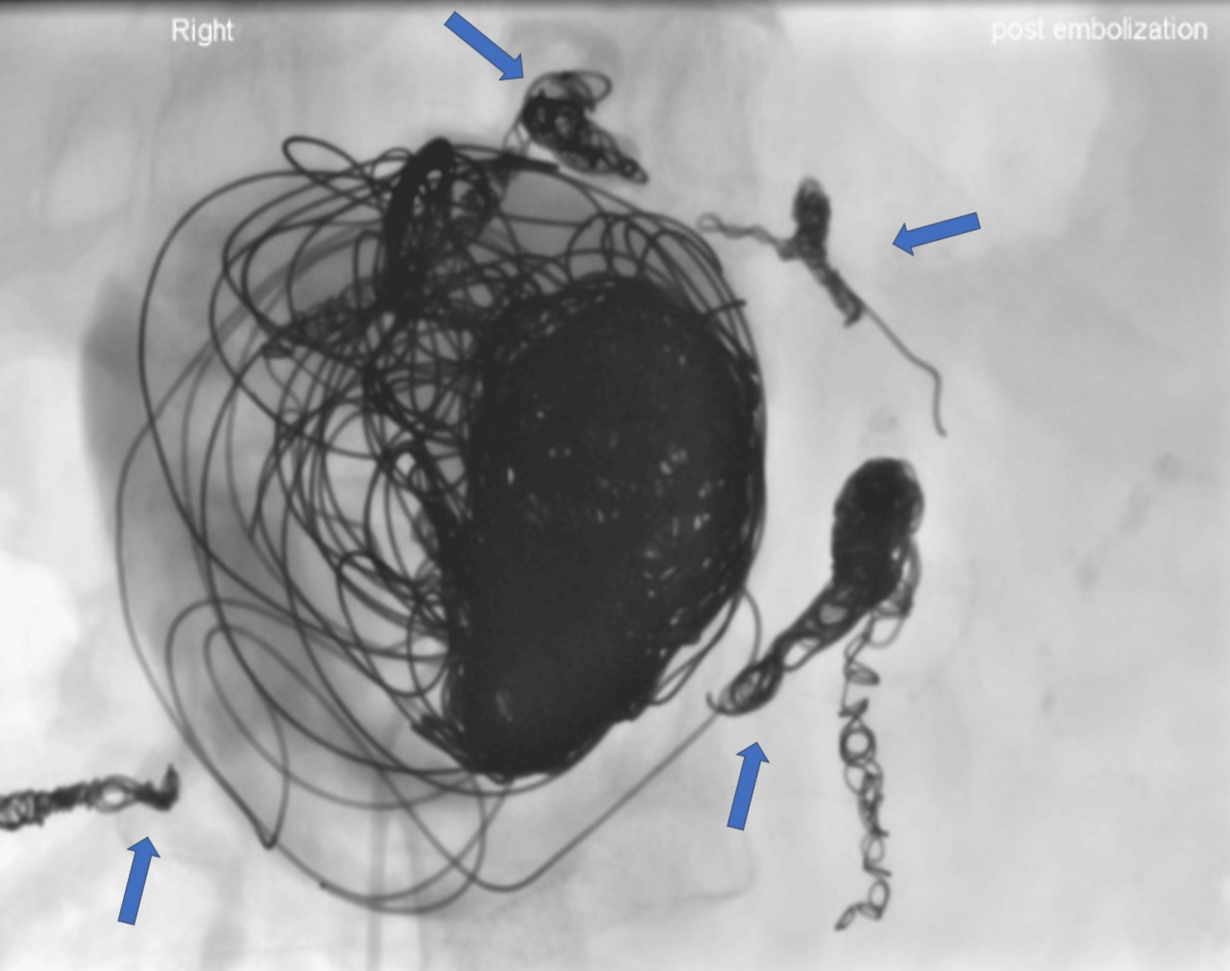
Haemorrhagic PP managed with multi-session angiographic embolization to achieve haemostasis Post-embolization direct angiography imaging showing PP with no further filling, as subsequently confirmed with CT. All adjacent feeding vessels embolised (blue arrows). PP: pancreatic pseudocyst; CT: computed tomogrpahy

Despite these measures, USS assessment showed persistent PP perfusion. A second direct transabdominal puncture was performed, and the cavity was embolized using a total of 21 detachable coils, including framing coils, standard detachable coils, and 60 cm soft packing coils. Residual peripheral pooling of contrast was identified and treated with a further direct thrombin injection of 4.000IU. Final angiography confirmed complete stasis without residual filling (Figure 5). The sheath tract was sealed using a microfibrillar collagen hemostatic agent and contrast mixture.

**Figure 6:**
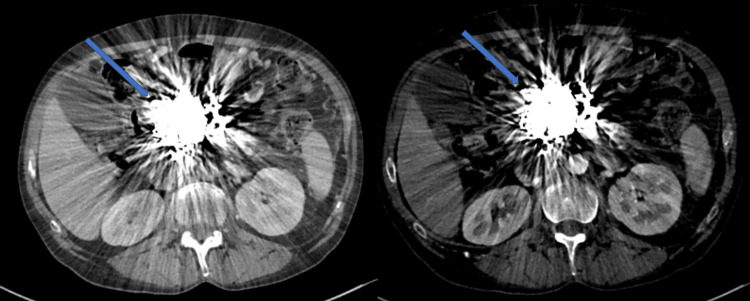
Haemorrhagic PP managed with multi-session angiographic embolization to achieve haemostasis Final post treatment appearances. Streak artefact from the coils makes assessment challenging (blue arrow), but no enhancement of the PP is appreciable on the arterial (left) or venous (right) phase on CT imaging. PP: Pancreatic Pseudocyst; CT: computed tomography

A follow-up CT scan performed 48 hours after the final intervention confirmed successful embolization of the entire PP with no further contrast opacification (Figure 6). The patient remained haemodynamically stable throughout his admission and received blood transfusions as clinically indicated. His liver function tests and renal function were regularly monitored and gradually improved (Table 1). He was discharged four weeks later on dual antiplatelet therapy.

**Table 1:** Serial blood tests results following sequential embolization procedures. Blood parameters are shown at key time points: after the third, fourth, and fifth embolization, on discharge, and at follow-up. WCC: white cell count; Hb: hemoglobin; Cre: creatinine; eGFR: estimated glomerular filtration rate; Bili: bilirubin; AST: aspartate aminotransferase; GGT: gamma-glutamyl transferase; ALP: alkaline phosphatase; INR: international normalized ratio

	3^rd^ Embolization	4^th^ Embolization	5^th^ Embolization	Discharge	Follow-up	Reference Range
WCC	4.2	5.2	8.0	6.9	4.0	2.9-9.6 10*9
Hb	77	90	85	90	110	115-135g/L
Cre	242	140	100	97	66	62-115μmol/L
eGFR	27	53	78	>90	>90	>90
Bili	5	8	<3	5	6	1.7-20 umol/L
AST	16	91	256	270	20	10-35 IU/L
GGT	188	477	937	1017	1353	5-40 IU/L
ALP	83	166	379	500	507	30-110 IU/L
INR	0.9	0.9	0.9	1.0	1.0	0.9-1.5
Albumin	32	32	31	34	35	35-50g/L

At outpatient follow-up eight weeks post-discharge, he was clinically well. Surveillance CT imaging confirmed complete resolution of the PP with no evidence of residual filling or secondary complications involving the bowel or liver.

## Discussion

Chronic pancreatitis is a condition characterized by recurrent inflammation of the pancreatic tissue leading to irreversible parenchymal destruction with fibrosis and atrophy [6]. Alcohol abuse is the predominant underlying cause, implicated in up to 77% of cases [7] and acute-on-chronic pancreatitis episodes are often [7].

The prolonged inflammatory course of chronic pancreatitis, combined with the anatomical proximity of adjacent vascular structures and necrotic pancreatic tissue, increases the risk of PA formation, seen in 4-10% of chronic pancreatitis cases compared to 1-6% in acute pancreatitis [8,9]. PAs are symptomatic in approximately 7.5% of cases [10], and presentations may range from mild abdominal discomfort or gastrointestinal bleeding to severe abdominal pain due to spontaneous rupture into the peritoneal cavity or a pre-existing pseudocyst [8,10].

Haemorrhagic PP occurs in 3.2-10% of acute or acute-on-chronic pancreatitis cases [2,6,11,12]. The most commonly involved arteries include the splenic artery, gastroduodenal artery, and branches of the pancreaticoduodenal arcade, given their close proximity to the pancreas [8]. However, PAs can arise from any branch of the visceral arterial system [10].

The diagnosis of PA has been revolutionized by contrast-enhanced CT now being the diagnostic gold standard. Endovascular embolization has replaced surgery as the preferred approach, making previously high-risk surgical interventions largely obsolete [9].

The success rate of endovascular treatment is 96%, as established by a systematic review and meta-analysis [9]. The choice of embolic agent depends on several factors, including the size and neck of the pseudoaneurysm, the parent artery, its anatomical location, and the patient’s coagulation profile. Surgery is now reserved for patients in whom embolization has failed, bleeding has recurred, or embolization is not a viable option [4,13].

Glubran II is a synthetic cyanoacrylate-based surgical glue that polymerizes rapidly upon contact with blood, forming a strong and durable embolic cast. It is often mixed with Lipiodol to modulate polymerization time and radiopacity, with common dilution ratios ranging from 1:1 to 1:3 depending on flow dynamics and target vessel anatomy. Glubran II has demonstrated efficacy in embolizing visceral PAs, particularly in high-flow or complex lesions where conventional agents such as coils may be insufficient or pose risk of migration [14-16].

In PAs associated with pancreatitis, where tortuous vessels and collateral supply are frequent, Glubran II offers superior penetration and durable occlusion [17]. Moreover, its use in combination with coils (the “sandwich technique”) or in repeated interventions allows tailored embolization strategies in refractory cases, as illustrated in our patient. Multiple studies have also shown Glubran II to be safe and effective in the management of bleeding complications across a range of gastrointestinal and vascular emergencies [15,18]. Its role in IR continues to expand as operator experience and technique-specific refinements evolve.

In our patient, the presence of decompensated liver cirrhosis and ascites rendered surgery extremely high risk. Any operative intervention, particularly pancreatic resection, would have carried a high risk of intraoperative bleeding and postoperative morbidity. If surgery were considered, total pancreatectomy would likely have been favoured over pancreaticoduodenectomy due to the risk of an anastomotic leak and the hostile local tissue environment. Given the increased morbidity and mortality associated with surgical treatment in such complex cases, a repeat endovascular approach was deemed safer and ultimately successful, facilitated by the availability of advanced interventional radiology resources in our tertiary care centre.

Several other documented cases highlight the need for repeat angiographic interventions to control persistent or recurrent bleeding [19-21]. Numoto et al. in a series of 19 PAs associated with pancreatitis, reported a 16% recurrent bleeding rate after initial Lipiodol or coil embolisation, some of which required repeat IR sessions for definitive haemostasis [21]. Overall, these cases reinforce that multi-stage endovascular strategies, including coil, glue, thrombin, and stent deployment, are critical tools for achieving sustained haemostasis in complex haemorrhagic pseudocyst scenarios.

Given the level of interventional radiology expertise required and the range of embolic materials employed, this case represents an extreme end of complexity in endovascular management of PA developed in the context of PP following pancreatitis. To our knowledge, it is the first detailed report of persistently haemorrhagic PP despite embolization of feeding vessels, managed successfully through a multi-stage IR approach. The extensive use of Glubran II, in combination with coils, thrombin, and covered stent deployment, underscores the critical role of advanced embolization strategies in high-risk patients. This case adds significant insight to the existing literature on complex visceral haemorrhagic PP management.

## Conclusions

This case highlights the complex and life-threatening nature of haemorrhagic PP complicated by PA, particularly in the setting of pancreatitis and decompensated liver disease. Despite multiple sources of arterial inflow and recurrent revascularization of the PP, definitive resolution was achieved through a staged, multidisciplinary IR approach, avoiding high-risk surgery. The patient's clinical stability and favorable outcome underscore the pivotal role of IR as a primary treatment modality in high-risk vascular complications.

Timely referral to specialized centres with advanced imaging, endovascular tools, and coordinated multidisciplinary care proved critical. To our knowledge, this is the first reported case of a persistent haemorrhagic PP treated successfully with a multi-stage IR strategy demonstrating an effective approach in managing complex pancreatitis-related emergencies.
